# Cervical cell’s nucleus segmentation through an improved UNet architecture

**DOI:** 10.1371/journal.pone.0283568

**Published:** 2023-10-03

**Authors:** Assad Rasheed, Syed Hamad Shirazi, Arif Iqbal Umar, Muhammad Shahzad, Waqas Yousaf, Zakir Khan

**Affiliations:** Department of Computer Science & Information Technology, Hazara University Mansehra, Mansehra, Pakistan; VIT-AP Campus, INDIA

## Abstract

Precise segmentation of the nucleus is vital for computer-aided diagnosis (CAD) in cervical cytology. Automated delineation of the cervical nucleus has notorious challenges due to clumped cells, color variation, noise, and fuzzy boundaries. Due to its standout performance in medical image analysis, deep learning has gained attention from other techniques. We have proposed a deep learning model, namely C-UNet (Cervical-UNet), to segment cervical nuclei from overlapped, fuzzy, and blurred cervical cell smear images. Cross-scale features integration based on a bi-directional feature pyramid network (BiFPN) and wide context unit are used in the encoder of classic UNet architecture to learn spatial and local features. The decoder of the improved network has two inter-connected decoders that mutually optimize and integrate these features to produce segmentation masks. Each component of the proposed C-UNet is extensively evaluated to judge its effectiveness on a complex cervical cell dataset. Different data augmentation techniques were employed to enhance the proposed model’s training. Experimental results have shown that the proposed model outperformed extant models, i.e., CGAN (Conditional Generative Adversarial Network), DeepLabv3, Mask-RCNN (Region-Based Convolutional Neural Network), and FCN (Fully Connected Network), on the employed dataset used in this study and ISBI-2014 (International Symposium on Biomedical Imaging 2014), ISBI-2015 datasets. The C-UNet achieved an object-level accuracy of 93%, pixel-level accuracy of 92.56%, object-level recall of 95.32%, pixel-level recall of 92.27%, Dice coefficient of 93.12%, and F1-score of 94.96% on complex cervical images dataset.

## Introduction

Cervical cancer often occurs in the cervix, a narrow cylindrical path of the lower uterus known as the neck of the womb. Cervical cancer is the world’s fourth most prevalent cancer, accounting for roughly 25000 fatalities among women per year [1]. This rate has been on the decline since 1950, attributed to the availability of different screening tests. Over the past four decades, histopathology imaging has been the gold standard in all forms of cancer investigations. Digital histopathology images are acquired from Image capturing, tissue slicing, staining, and digitization, where the images generally have big resolutions. Many nuclei are often found in a tissue slide with varying shapes, appearances, textures, and morphological features. The primary analytical procedures in digital histopathology are the segmentation of nuclei from cells, glands, and tissues. The segmented nuclei provide indicators that are critical for cancer diagnosis and prognostic. A simplified Papanicolaou (Pap) smear test, thinPrep Cytology test (TCT), and liquid-based cytology (LBC) are the most commonly used screening methods to detect cervical cancer [2–4]. The cytological scans of the cervical smear are investigated at 400 x magnification through the microscope. With this magnification, pathologists have to examine multiple field-of-views per scan, which takes a lot of time, extremely susceptible to errors and observer bias. This process becomes more difficult due to cell clumps, yeast contamination, or bacteria masking by blood, mucus, and inflammation.

Several automated diagnostic methods have been designed to help cytologists to examine viginal smears of Pap strains, which are discussed in related work. A number of factors continue to pose a challenge to this task, including the presence of overlapping nuclei, superficial cells, poor contrast, spurious edges, poor staining, and cytoplasm. Therefore, more robust automatic screening systems are required to assist cytologists in determining cytopathy in cervical cells. A vital component in the Cervical Cytology pipeline is the precise segmentation and detection of a nucleus from Cervical cells [5]. An increasing number of studies primarily intrigued the delineation of cells cluster and nuclei. Watershed [6], morphological operation, thresholding [7], and active contour models [8] are a few of the many methods employed for the segmentation of nuclei or cellular mass. These techniques failed to delineate overlapping cell structures well.

**Fig 1:**
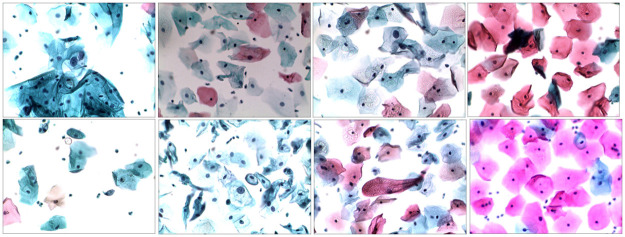
Shows cervical cell images from the employed dataset.

Recent studies [9] have shown some improvements in the isolation of clumping nuclei and cytoplasm from cervical cells. The datasets used in most of these studies for the delineation of nuclei include the liquid Based Cytology (LBC) Pap smear dataset and Herlev dataset for single-cell delineation and Shenzhen University (SZU), ISBI2014, and ISBI2015 datasets for multi-cell examination. These datasets images show nuclei with isolated, apparent color variation and separated boundaries among cell constitutes, making analysis easy. According to [10], despite good performance on those datasets, several approaches mentioned above failed to excel in their dataset based on real-world clinical data having folded nuclei, cytoplasm, and color differences. Similarly, the majority of prior studies [11–14] focused on the segmentation of huddled cytoplasm, and a few [15–17] concentrated on the delineation of nuclei from those datasets. Recently, deep neural networks [18–20] have surged in popularity for their standout performance in the field of medical image analysis. Deep learning models can handle complex tasks if the size of the datasets is big enough [21]. Presently, datasets in hand possess lesser diversity of nuclei shape, appearance, and color than real-world clinical data. We have selected a challenging dataset consisting of 104 cervical cell images of size 1024 x 768 based on LBC screening. Fig 1 shows a few examples of cervical images having variations in shape, texture, color, and appearance, making the dataset more challenging. Furthermore, the continuous pooling and convolution operations across the network result in the loss of vital information required for the precise segmentation of nuclei.

To handle these issues, effective feature extraction, and adaptation to the heterogeneity of cervical cells, an improved UNet model with BiFPN is suggested, which is suitable for segmenting various forms of cervical cell nuclei. Overall, the contributions of this study are listed in the following:

1. We have designed an optimized network based on UNet architecture, namely C-UNet, for precisely segmenting nuclei from cervical images.
2. C-UNet strategically integrates the Cross-scale features (CSFI) module in C-UNet to integrate features extracted by the network using a cross-scale weighted integration scheme into the final feature map. This fusion adaptively combines the elements of different spatial resolutions and domains based on their significance.
3. C-UNet uses two interconnected decoders for boundary detection and semantic segmentation of cervical nuclei.
4. To evaluate the usefulness of each part of C-UNet multiple experiments were performed.

The remainder of this manuscript is structured as follows: related work is illustrated in section 2, section 3 describes the architecture of the suggested model, followed by results and discussion in section 4, and the conclusion is provided in section 5.

## Related work

A slew of successful cytological nuclei segmentation methods has been developed during the previous two decades. Prior techniques for segmenting cytological nuclei can be categorized as handcrafted and deep learning techniques.

### Handcrafted methods

Traditional methods for cytological nuclei segmentation are based on edge enhancement [22], thresholding [17, 23–25], clustering [8, 9, 26, 27], morphological features and marker controlled watershed [28, 29]. [22] provides a cervical nucleus and cytoplasm detector based on edge enhancement. Depth equalization was employed to enhance edges but failed to segment blurry contours. [17] developed a model for the locating and segmentation of nuclei from cytological images. A randomized Hough Transform with prior knowledge is used to locate nuclei, and a level-set segmentation method is used to separate nuclei from cell regions. Otsu thresholding, median filter, and canny edge detector were employed during preprocessing stage. Such techniques are not applicable to the splitting of crowded cell structures.

[6] proposed thresholding for separating cell regions and multiscale hierarchal method based on circularity and homogeneity segmented regions. A binary classifier was used to distinguish cytoplasm from the nucleus. The ranking of the cells was determined by linearizing the leaves of a binary tree created using hierarchical clustering. Cases with irregular sizes and shapes were poorly handled. [25] designed an automated method to segment nuclei from cervical cells. They employed V-channel to enhance the contrast between cytoplasm and nuclei. Two features, roundness and shape factor, were used to validate the segmentation of clumped nuclei. Concave-point algorithms were applied to segment multi-clumped nuclei. To remove noise and non-uniform illumination, median filter and adaptive thresholding were used. Zhao et al. [27] addressed the problem of cervical cell segmentation via Markov Random Field (MRF) based on superpixel. A gap search method was applied to minimize time complexity. Sahe et al. [9] utilized a superpixel merging approach for accurate segmentation of crowded cervical nuclei. The superpixel is obtained through the statistical region merging (SRM) technique through the pair-wise regional control threshold of SLIC (Simple Linear Iterative Clustering) superpixel. This technique could not resolve the under-segmentation issue and failed to tackle the heterogeneity of nucleus size and shape.

Morphological analysis and thresholding-based approaches have been used for the crisp segmentation of cervical nuclei. [28] introduced an iterative thresholding technique to segment nuclei on the basis of the size of the nucleus, solidity, and intensity. [23] employed local thresholding to segregate cervical nuclei on the basis of properties within a window of a radius of 15 pixels. In [24], traditional Otsu thresholding was integrated with contrast-limited adaptive histogram and anisotropic filtering to detect nuclei. Thresholding-based methods do not work well for intractable cases. In [7, 30], morphological features were used to locate the centroid of the nucleus, which is subsequently employed to determine the boundaries of the nucleus. [30] used centroid to determine markers for watershed transform, and [7] utilized them to find radial profiles. These techniques were unable to segment varying shape cellular structures. In [31], an adaptive local graph-cut approach for cervical cell delineation was applied with a blend of area information, texture, intensity, and boundary. Graph-based method’s performance degrades in the case of touching objects.

Watershed is a prevailing image segmentation technique that has been around for a long time. Several studies [12, 32] have determined that marker-controlled watershed segmentation is effective. [12] developed a multi-pass watershed algorithm with barrier-based watershed transform for cervix cell segmentation. The first finds the nucleus on a gradient-based edge map; the second pass segregates touching, isolated, and clumped cells, and the third pass estimates the cell shape in the clumped clusters. These methods are susceptible to noise and need pre and post-processing, which is quite cumbersome.

These approaches have the apparent flaw of not being able to adequately split cervical nuclei since they frequently rely on an incomplete set of low-level hand-crafted features. Additionally, these features lack structural information and yield below-par segmentation results. Thus, various pre and post-processing steps are needed for different approaches for various types of nuclei to enhance the segmentation quality. However, the lengthy pipeline and complicated, intricate process flow often encounter instability. The entire segmentation procedure might fail if errors occur in the intermediatory phases.

### Deep activated features based methods

Deep learning-based models are one of the most recent advances in numerous applications and are widely employed in cytological image analysis. [33] integrated a superpixel with a Convolutional neural network (CNN) for precise segmentation of nuclei and cytoplasm from cytological images. A trimmed mean filter was used to remove noise from input images. CNN was used to learn 15 features from superpixels. Coarse nucleus segmentation was performed to decrease the clustering of inflammatory cells. The nuclei segmentation accuracy was noted as 94.50%. Coarse segmentation becomes inconsistent for cases when the receptive field is larger than the nuclei. In [34], a fully convolutional network (FCN) was employed to separate the nucleus region, and a graph-based method was used to obtain finer segmentation. They recorded an accuracy of 94.50% on a dataset comprising 1400 images. FCN employs single-scale features that produce inconsistent results for complex cases. Song et al. [35] investigated a multiscale convolutional network (MSCN) with graph partitioning via superpixel for cervical nuclei and cytoplasm segmentation. The results demonstrated that MSCN, graph partitioning, and superpixel effectively delineate cervical cells. Superpixel segmentation has shown an increase of 2.06% for the nucleus and 5.06% for the cytoplasm compared to raw pixels segmentation. However, the employed method cannot detect isolated and touching nuclei in the same process. Phoulady et al. [36] used an iterative thresholding method with CNN for the cervical nucleus delineation model. They trained the model using nuclei patches of size 75 x 75 pixels. This method achieved a recall, precision, and F-score of 86%, 89%, and 87% on a complex CERVIX93 dataset. This method is not able to isolate touching cellular nuclei boundaries. Liu et al. [37] designed a model based on Mask-Regional Convolutional Neural Network (Mask-RCNN) and Locally Fully Connected Conditional Random Field (LFCCRF) models. The Mask-RCNN incorporates multiscale feature maps to perform accurate nuclei segmentation, and LFCCRF uses abundant spatial information to refine the nucleus boundary further. Experimental results on the Herlev Pap smear dataset show superior performance compare to most of the prior studies. Conditional Random Field based methods employ second order statistics whilst higher order statistic is more beneficial for segmentation of images. In [38], two functional extensions were introduced in Faster RCNN, i.e., global context aware function to improve spatial correlation and deformable convolution function in the last three layers of FPN (feature pyramid network) to enhance scalability. Experiments have shown a reasonable improvement of 6–9% on mAP (mean average precision) on the cervical images dataset. RCNN is not able to delineate nuclei having varying aspect ratio and spatial locations.

Presently, there are few models developed expressly for the detection of cervical cancer [39–41]. Tan et al. [39] deployed a faster RCNN for the identification of cancer cells in TCT images and obtained an AUC of 0.67. Zhang et al. [42] investigated R-FCN architecture for cancer cell detection in LBC images. This architecture focuses on detecting the abnormal region instead of abnormal nuclei. The performance was evaluated on a novel notion termed hit degree, which defines how closely each ground truth and detected region are from each other. Li et al. [43] introduced a faster RCNN to identify and classify abnormal cells in cervical smear images scanned at 20 x. In [44], the authors presented a generative adversarial network (GAN) to successfully segment both overlapping and single-cell images. The proposed GAN used structural information of the whole Image and the probability distribution of morphology of the cell for segmentation. Compared to other state-of-the-art models, results show good performance on poor contrast and highly overlapping cells. The model produced significant DC (dice coefficient) and FNRo (false negative rate) values of 94.35% and 7.9% for single cells, 89.9%, and 6.4% for clumped cells. The GAN-based models are very difficult to train and unsuitable for small datasets. Chen et al. [45] used two staged Mask-RCNN to segment cervical cytoplasm and nuclei. ISBI-2014 and ISBI-2015 were used to evaluate the model and witness increased performance for segmenting cervical cellular masses. Yang et al. [46] applied a deep learning model based on a modified UNet. They used ResNet-34 as an encoder block leveraged with Gating Context-Aware Pooling layers to extract and refine learned features. The modified decoder uses a global attention layer to build long-range dependencies. The proposed model was trained and tested on a private dataset, namely the ClusteredCell dataset. Results show significant improvement in the performance of their model compared to state-of-the-art (SOTA) models. [47] employed UNet embedded local and global attention layers. These multi-attentions layers enhance the network capabilities to extract and utilize features for segmentation of cervical cytoplasm and nucleus. The Herlev dataset was used to evaluate model performance and recorded better segmentation scores than SOTA models. [48] designed a deep learning-based model for the segmentation of nuclei from multiple datasets. Tissue-specific features were extracted from histopathological images using BiFPN. Post-processing steps are required to further improve output. Excellent segmentation results were recorded as compared to other benchmarking networks. [49] developed a 3 phase cervical cell segmentation method. In the first phase, a CNN is used for coarse segmentation of cellular masses; the second phase identifies the location of cytoplasm and nuclei with a random walker graph; the third phase refines the output of previous phases through the Hungarian algorithm. A DSC score of 97.2% was noted for the ISBI-2014 dataset. [50] proposed a framework based on an adversarial paradigm for spotting the cervical cell. They used RCNN to construct the encoder & decoder and fine-tuned (FSAE) autoencoder to optimize parameters. Analysis of results shows an improvement in the performance of the proposed framework. [51] proposed Triple-UNet and exploited Haemotoxylin staining to predict cellular nuclei. They subtracted boundaries from the segmentation map to split clumped nuclei. The drawback of this technique is that such subtraction of instance boundary may reduce segmentation accuracy. [52] designed a model, StarDist, to estimate the centroid probability map and distance from each foreground pixel to its corresponding instance boundary. Each polygon map corresponds to one nuclear instance. This technique only predicts polygons based on the centroid pixel’s characteristics, which lacks contextual information for big-sized nucleus occurrences, which lowers segmentation accuracy.

## Material and method

### C-UNet’s architecture

#### Overview

**Fig 2:**
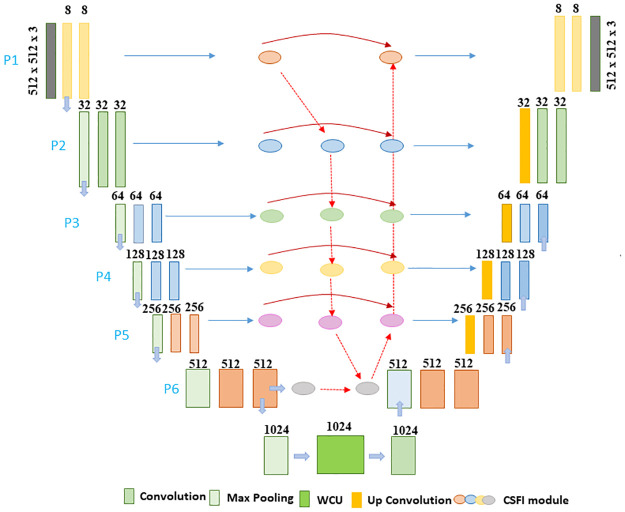
Presents a structure of the proposed C-UNet model.

The complete structure of the C-UNet is presented in Fig 2, comprising three modules, i.e., encoder, Cross-scale features integration (CSFI), and interlinked decoder. The encoder learns the nuclei features, which are passed on to the CSFI module to generate rich and precise feature representation. This benefits the inter-connected decoders to create valid and reliable activations for each nuclei sample.

#### UNet architecture

We have suggested a cervical cell segmentation architecture based on U-Net, composed of a multiscale feature extractor encoder and inter-connected decoders. The network takes 512 x 512 x 3 image as input and output a 512 x 512 mask. The encoder has the classic architecture of a convolution network. It comprises two successive convolutions of 3 x 3 with similar padding followed by Rectified Linear Unit (ReLU) activation function and maximum pooling of 2 x 2 with a stride of size 2 for downsampling. With each downsampling, the number of feature channels is doubled. The levels of the encoder are seven. The features at the sixth level fed into the CSFI unit and WCU simultaneously, and output fed into the interlinked decoders. For regularization of the model, a dropout layer is used with a factor of 0.5. Each level in the encoder is composed of an upsampling of the feature preceded by the convolution layer of size 2 x 2, which reduces the number of features at each level to half. The extracted features due to upsampling are integrated with the analogous features from the feature network. The integration is followed by two convolutions of 3 x 3 with the same padding, each preceded by the ReLU function. In the last layer, the acquired 512 x 512 x 64 features go through two convolutions of 3 x 3. It is preceded by the ReLU function, the last convolution operation of 1 x 1, and the sigmoid function. In the final layer of the backbone network, the obtained 512 x 512 x 64 feature map undergoes two 3 x 3 convolutions.

#### Cross-scale features integration (CSFI) module

**Fig 3:**
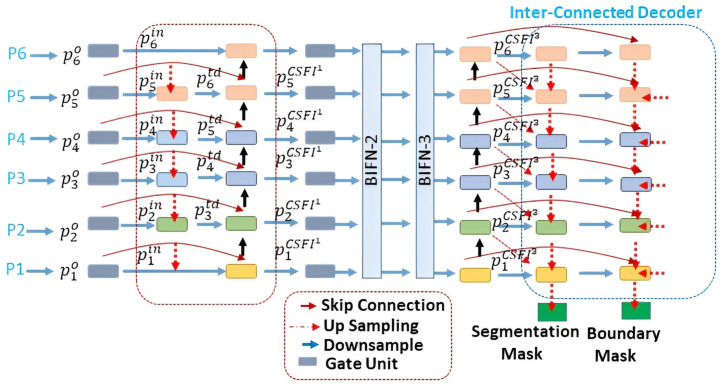
Shows the cross-scale feature integration module, inter-connected decoders, and its essential components.

We have used the UNet model to extract features from input images in different levels with the help of Convolution layers. To minimize the noise response, computation overhead and to concentrate on specific features, we have employed a gating mechanism. The gating unit surpasses the response of insignificant regions and focuses on nuclei features. It is composed of 1 x 1 convolution, batch normalization, sigmoid activation, and dropout function. The CSFI comprises multiple bottom-up and top-down pathways based on insight from BiFPN [53], as shown in Fig 3. These pathways merge low and high-level features in an efficient way. Output from multiple levels of the UNet was employed as input to the BiFPN model for feature integration. The BiFPN model outputs the features on seven different levels ranging from *p*_1_ to *p*_7_.

Formally, given a set of multi-level features ($p_{l}=p_{1}^{i},p_{2}^{i},\ldots p_{7}^{i}$) at *i*^*th*^ level and $p_{l}^{in}=p_{1}^{in},p_{2}^{in},\ldots p_{7}^{in}$) represents an intermediate feature set on the top-down path. The objective is to aggregate multiscale features to get $p_{l}^{o}=\int (p_{l}^{in})$. We have employed a weighted integration mechanism to fuse features of various resolutions. The integration mechanism is mathematically shown in Eq 1.

$$I=\sum_{i}\frac{v_{i}}{e+\sum_{j}v_{j}}$$ (1)

*v*_*i*_ represents extra learnable weight, ReLU is employed to ensure that the value of additional weight should be greater than or equal to zero and the value of *e* set to 0.0001. This enables to rectify the relevance of each channel of the input representation.

As illustrated in Fig 3. The intermediate fused features maps are calculated using Eqs 2 and 3 as follows

$$p_{l}^{o}=Convolution\left[\frac{v_{i}.\;p_{l}^{in}+v_{i+1}.\;R_{b}\left(P_{l+1}^{o}\right)}{v_{i}+v_{i+1}+e}\right],$$ (2)

Where $p_{l}^{o}$ represents intermediate feature maps of *i*^*th*^ level on the top-down path, *v*_*i*_ shows the weighting vector, $p_{l}^{in}$ is the input features vector. Intuitively, *v*_*i*_ shows the significance of the features map $p_{l}^{in}$. If $p_{l}^{in}$ is crucial to the output, then *v*_*i*_ will be assigned a bigger value during training.

$$p_{l}^{CSFI^{1}}=Convolution\left[\frac{v_{i}.\;p_{l}^{in}+v_{i}^{\prime }*p_{l}^{o}+v_{i}^{\prime }.R_{b}\left(P_{l-1}^{CSFI^{i}}\right)}{v_{i}^{\prime }+v_{i+1}^{\prime }+e}\right],$$ (3)

$P_{l-1}^{CSFI^{1}}$ indicates the output of the CSFI module, *R*_*b*_ symbolizes bilinear interpolation to resize feature maps before adding at pyramid level *l* and *i* indicates a series of CSFI modules, i.e., 3 in our case.

#### Decoder

The Features extracted by the CSFI module are passed into the decoder. The decoder module jointly integrates multiple-scale features from CSFIs and generates precise segmentation and boundary masks. After resizing by bilinear interpolation *R*_*b*_, the optimized feature maps from *i*^*th*^ CSFI module passed through the feature integration unit. This iterative procedure is performed until the final masks at the (L-1) pyramid level are generated. For instance, segmentation and boundary maps at 3^*rd*^ level is computed as shown in Eqs 4 and 5 below

$$P_{3}^{seg}=Convolution\;\left[v_{CSFI^{3}}^{1\;x\;1\;x\;c_{3}}.\;p_{3}^{CSFI^{3}}+v_{seg}^{1\;x\;1\;x\;c_{4}}.R_{b}\left(P_{4}^{seg}\right)+v_{CSFI^{3}}^{1\;x\;1\;x\;c_{4}}R_{b}\left(\;p_{4}^{CSFI^{3}}\right))\right]$$ (4)

Where $P_{3}^{seg}$ symbolizes feature maps at 3^rd^ level. $v_{CSFI^{3}}^{1\;x\;1\;x\;c_{3}}$ represents features maps obtained at the 3^rd^ pyramid level, $p_{3}^{CSFI^{3}}$ shows reweighted features maps of 3^*rd*^ CSFI module, $p_{3}^{CSFI^{3}}$ are the features obtained after filtration through the gating block.

$$P_{3}^{bou}=Convolution\left[v_{seg}^{1\;x\;1\;x\;c_{l}}.\;p_{l}^{CSFI^{l}}+v_{bou}^{1\;x\;1\;x\;c_{l+1}}.R_{b}\left(P_{l+1}^{bou}\right)+v_{CSFI^{l}}^{1\;x\;1\;x\;c_{l+1}}R_{b}\left(\;p_{l+1}^{CSFI^{l}}\right))\right]$$ (5)

$P_{3}^{bou}$ indicates boundary detection maps at 3^rd^ level. A refinement unit is incorporated to refine feature maps further. It comprises two convolutional layers of kernel size 3 x 3 and 1 x 1, respectively.

#### Wide Context Unit (WCU)

**Fig 4:**
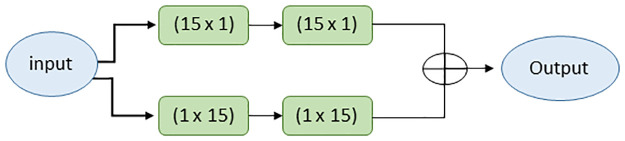
Structure of WCU.

WCU, like the CSFI module, learns the contextual information and performs feature accumulation at the transition level, enabling improved reconstruction of segmented nuclei. The WCU consists of two parallel connections of two convolutional layers with different combinations, and the output from them is added up to an output vector, as shown in Fig 4. The convolution layers in the first connection have filter sizes of N x 1 and 1 x N, respectively. Similarly, convolution layers in the second connection have filters of dimensions 1 x N and N x 1.

### Loss functions

The loss function or cost function is used to evaluate the prediction of the network. The lower the cost function value, the higher the model performance. We have employed multiple loss functions to evaluate the performance of the proposed model.

#### Baseline module loss

We have used a weighted summation of three-loss functions for efficient convergence and speedy training of the suggested model, i.e., dice loss, binary cross-entropy, and focal loss. The respective mathematical dynamics are expressed in Eq 6 below:

$$loss_{seg}=w_{seg}\left(loss_{Dice}+Loss_{BCE}\right)+loss_{ft}$$ (6)

Where *loss*_*Dice*_ represents dice loss, *Loss*_*BCE*_ indicates binary cross-entropy loss and *Loss*_*ft*_ shows a focal loss. *w*_*seg*_ symbolizes the hyper-parameter that regulates dice and binary cross-entropy losses.

Numerically respective losses are expressed in Eqs 7, 8 and 9 below

$$loss_{Dice}=1-\frac{2\;x\;\sum_{i=1}^{P}\;\rho_{ic}L_{ic}+E}{\sum_{i=1}^{P}\;P_{ic}+\sum_{i=1}^{P}\;L_{ic}+E}$$ (7)

$$loss_{BCE}=-\frac{1}{n}\;\sum_{i=1}^{P}\;\sum_{c=1}^{c}\;\rho_{ic}\;\text{log}\left(L_{ic}\right)$$ (8)

$$loss_{FT}=\sum_{c}{\left(1-\frac{\sum_{i=1}^{P}\;\rho_{ic}L_{ic}+E}{\sum_{i=1}^{P}\;P_{ic}L_{ic}+\alpha \;\sum_{i=1}^{P}\;\rho_{ic}L_{ic}+\beta \;\sum_{i=1}^{P}\;\rho_{ic}L_{ic}}\right)}^{\frac{1}{\propto }}$$ (9)

Where *P* symbolizes the total number of pixels, *c* indicates classes, and E represents the smoothness constant. *ρ*_*ic*_ and *L*_*ic*_ shows probability of *i*^*th*^ pixel of class and ground-truth label. *α*, *β*, and ∝ denotes hyper-parameters in the focal loss function.

#### Boundary detection loss

We have employed a combination of combo loss and focal loss, which is mathematically expressed in Eq 10 as

$$Loss_{bou}=loss_{ft}+w_{bou}\left(loss_{com}\right)$$ (10)

Where *Loss*_*bou*_ represent boundary loss and *Loss*_*com*_ shows combo loss mathematically expressed in Eq 11.

$$loss_{com}=\alpha \left(-\frac{1}{P}\sum_{i=1}^{P}\;\beta \left(L_{ic}-\text{ln}\left(\rho_{ic}\right)\right)+\left(1-\beta \right)\left[\left(1-L_{ic}\right)\text{ln}\left(1-\rho_{ic}\right)\right]\right)-\left(1-\alpha \right)\left(loss_{Dice}\right)$$ (11)

Where *P* number of pixels, *ρ*_*ic*_ and *L*_*ic*_ shows probability of *i*^*th*^ pixel of class and ground-truth label. *α*, *β*, and ∝ represents focal loss hyper-parameters.

## Experimental setup

### Datasets

Many cases in the clinical environment have cervical images with overlapped, self-folded, blurred contours, different sizes and shapes of nuclei, and color similarity with the cytoplasm. At the same time, few cervical scans have impurities, i.e., stains, illumination, and focus variabilities. To meet the practical requirements, the system should handle such cases properly. In deep learning, the size of labeled data is critical for satisfactory performance. It needs time and expertise in the medical field to properly build and annotate cervical cytology images. We have used the cervical cytology image dataset published by [54] for this research, which contains complex cases from the real-world clinical environment. Fig 1, 6 and 7 show some samples from the dataset. It is composed of 104 LCT cervical images of size 1024 x 768. Each instance has a ground truth marked very carefully by the experienced pathologist. The images are generated via Olympus microscope B x 51 with an adequate pixel size of 0.32μm x 0.32μm and 200x magnification.

To justify the performance of the C-UNet, two standard datasets (ISBI2014 and ISBI2015) were also used in this study. The ISBI2014 dataset consists of 45 training images and 900 test images generated synthetically with different cell counts and overlap ratios. The ISBI2015 dataset contains 17 samples, and each consists of 20 other (Extended Depth of Field Images) EDF from multiple focus planes in a field of view. Each Image comprises 40 cervical cells with various cell numbers, overlaps ratios, texture, and contrast. We have used eight images for training and 9 for testing purposes. Table 1 shows the number of images before and after data augmentation, and the impact of data augmentation on the model performance is reflected in Table 6.

**Table 1:** Shows the total number of images used in this study.

Datasets	No. of Images	No. of Images after Augmentation
LCT dataset used in this study	104	640
ISBI-2014	45	445
ISBI-2015	34	350

### Preprocessing

We applied data augmentation, boundary enclosing, and stain normalization to obtain better performance.

#### Boundary enclosing

**Fig 5:**
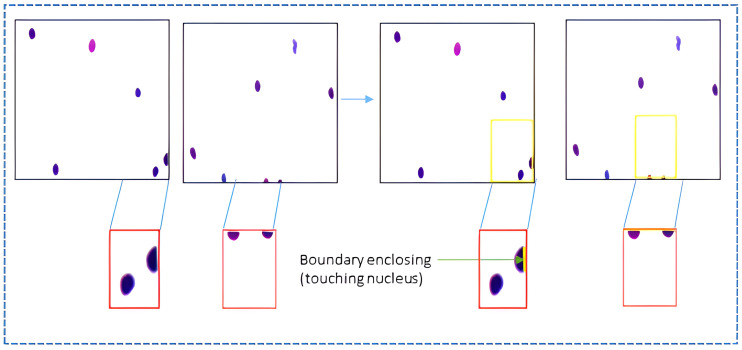
Shows the boundary enclosing of the cervical nuclei.

We have performed boundary enclosing of the nuclei at the edges of the image and patches of the cervical cells, as presented in Fig 5. The open boundaries at the edges bring inconsistency during training and may escalate further with data augmentation, i.e., scaling, rotation, resizing, cropping, and transformation. Boundary enclosing assists the model in identifying the nonexistent and fuzzy boundaries around the edge of cervical cell images.

#### Data augmentation

**Fig 6:**
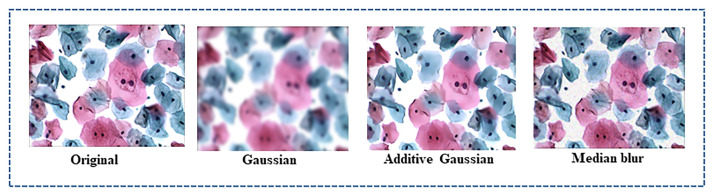
Shows cervical images after the addition of different types of noise.

**Fig 7:**
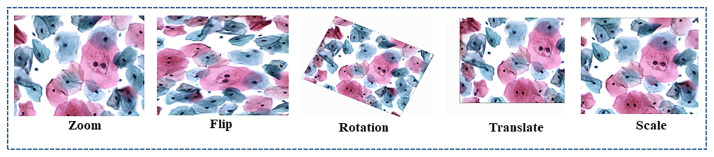
Shows different types of data augmentation for cervical images.

To minimize deep learning model generalization errors and enhance the model’s performance, we have performed data augmentation to produce new instance points in the cervical image dataset. The total number of images after data augmentation becomes 640. This prevents overfitting and regulates model training for a long period. We have employed two types of data augmentation techniques for the cervical cell dataset. Geometrical transformation (resizing, vertical and horizontal flipping, rescaling and resizing, image translation, and elastic transformation) is depicted in Figs 6 and 7 below. Noise and intensities transformation includes blur, Gaussian, and additive Gaussian noise, along with Hue, Saturation, and Contrast adjustments. The impact of augmentation on the C-UNet performance is shown in Table 6.

#### Stain normalization

**Fig 8:**
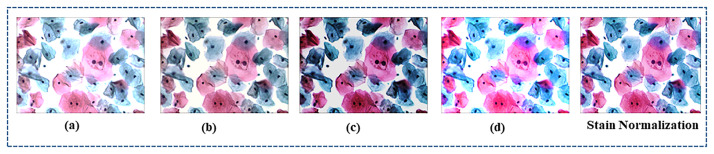
Shows the comparison of various image normalization approaches. Fig 8(a) represents the normalization method employed by [56], 8(b), and 8(c) shows the results of techniques designed by [57, 58].

The Hematoxylin and eosin (H & E) staining are extensively used in pathology to discriminate nuclei from cytoplasmic and other cellular structures. Hematoxylin is used to stain the nuclei of the cells, whereas eosin is used to stain the cytoplasm and extracellular matrix. This staining has color variation due to different response functions of scanner staining protocols and manufacturer design of stain vendors. These differences have undesirable effects on image interpretation of deep learning-based pathology because these methods mainly depend upon the texture and color of (H & E) images. The performance of deep learning models can be significantly improved by normalizing (H & E) images [55]. Nevertheless, normalization techniques designed for traditional computer vision applications provide inadequate benefits in computational pathology. Numerous techniques have been developed for standardizing pathology images [56–58]. [56] introduced an unsupervised learning method for stain density maps estimation by partitioning pathology images into sparse density maps and then integrating them with a stain-colored basis of the target image, as shown in Fig 8(a). This technique only changes the color and keeps the original Image’s structure. Our preliminary experiments suggested that sparse stain standardization performed better than standard normalization methods [57, 58] for cervical images, as shown in Fig 8.

### Training detail

Adopting the standards evaluation methods, we have split our cervical images dataset into training (80% of the whole dataset), evaluation (10% of the entire dataset), and testing (10% of the original dataset) sets. During the course of model training, stochastic gradient descent (SGD) was employed as an optimizer. The learning rate was set to 0.001 momentum of 0.9, and weight decay of 5e-4 was used to train the model. We have used the synchronous batch normalization (BN) method with a decay rate of 0.99. BN helps the model to converge early and minimize the chances of overfitting. ReLU activation function for the entire network and sigmoid activation function for the last layer with softmax. The batch size was set to 10, and constant smoothness rate was set to 1e-3, and an early stopping scheme was used to tackle overfitting. The description of the loss functions and their impact used are presented in Table 5 below. For training purposes, the original size of the images in the dataset was cropped to 256 x256 x 3 for augmentation. For testing, the image size was set to 512 x 512 x 3. The suggested model is trained on an Nvidia Ge-Force GTX 1080 GPU (graphical processing unit) with 12 GB of RAM. We have trained the C-UNet on a large cell nuclei dataset [59] and then retrained on our cervical cells dataset.

### Evaluation measures

To justify the performance of the proposed model on the cervical images dataset, we have employed accuracy, recall, and the Dice coefficient. For all these performance measures, the highest numbers represent the best results. The description of each of the criteria is shown below:

### Accuracy

Accuracy is the ratio of correctly identified cervical nucleus pixels to the total number of pixels in a cervical cytology image. Mathematically it is described as shown in Eq 12.

$$Accuracy=\frac{T_{P}+T_{N}}{T_{P}+F_{P}+F_{N}+T_{N}}$$ (12)

Where *T*_*P*_ symbolizes true positive, *T*_*N*_ shows true negative rates, *F*_*P*_ indicates false positive, and *F*_*N*_ represents false negative.

### Recall

The recall is measured as the ratio between the numbers of positive cervical nuclei pixels correctly identified as positive to the total number of pixels in the cervical cell image. Mathematically expressed in Eq 13 as

$$Recall=\frac{T_{P}}{T_{P}+F_{N}}$$ (13)

### Dice coefficient

The dice Coefficient is a statistical measure employed to determine the similarity of two instances. Mathematically it is expressed as in Eq 14.


$$D_{co}=\frac{2\;x\;area\;of\;overlap}{Total\;number\;of\;pixel\;in\;both\;images}$$ (14)

### F1-score

**T**he F1 is calculated as the ratio between precision and recall. The greater the F1 values, the better overlap between ground-truth and predicted cervical nuclei segmentation masks. Mathematically described in Eq 15.


$$F1-score=\frac{Recall\;x\;precison}{Recall+precison}$$ (15)

## Quantitative study and results

C-UNet consists of different image processing and deep learning approaches; therefore, we have extensively analyzed each module of the suggested deep learning network to comprehend how each part relates to performance improvement. We have performed five experiments under the same evaluation and training settings, and the results are given as the average of all experiments.

### C-UNet module

**Fig 9:**
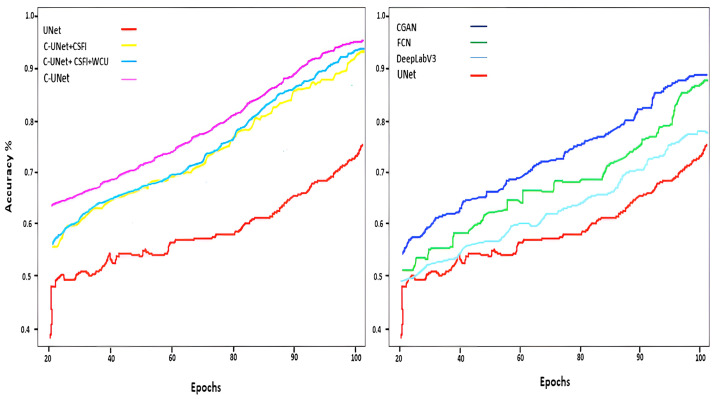
Models accuracy during training against epochs.

**Fig 10:**
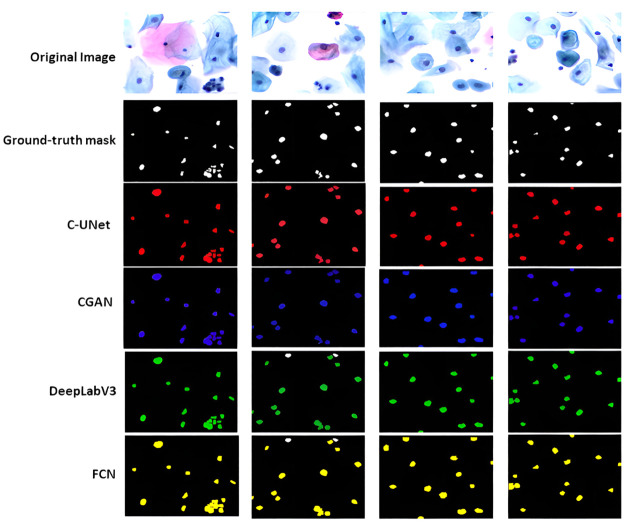
Shows cervical images and segmentation masks generated by state-of-the-art segmentation networks.

We have performed several experiments to explore the performance of different components in the C-UNet model. The impact of each element of the proposed model is shown in Table 2 and Fig 9. We have started with the standard UNet model, and the results are reflected in Table 2, row 1. In the second experiment, the CSFI module was incorporated into baseline UNet and noticed an improvement in the segmentation results. In the third experiment, we introduced a features reweighting unit and noticed a significant improvement in the segmentation performance of the proposed model. This is in line with our recommendation that integrating features from various domains and spatial resolutions retain local and spatial information across the pyramid. In the fourth experiment, we have incorporated WCU, which integrates features at the transitional level and helps model the precise reconstruction of segmented nuclei, as shown in Table 2 and Fig 10. Fig 8 shows original cervical cell images, ground truth, and prediction masks generated by benchmark networks and the proposed C-UNet.

**Table 2:** Performance comparison with benchmark architectures for cervical nuclei segmentation with C-UNet.

Method	Acc_o_	Recall_o_	Acc_p_	Recall_p_	Dice Coefficient	F1-Score
Standard UNet	73.21%	84.11%	80.33%	74.41%	85.21%	78.62%
C-UNet+CSFI	92.31%	94.32%	91.02%	89.92%	90.59%	93.98%
C-UNet+ CSFI+WCU	92.67%	94.96%	91.77%	91.13%	92.42%	94.71%
C-UNet+ CSFI+WCU+ ID	93.00%	95.32%	92.56%	92.27%	93.12%	94.96%
DeepLabv3	88.02%	86.11%	77.09%	84.21%	82.23%	88.17%
FCN	88.23%	91.33%	88.27%	83.03%	90.49%	84.52%
CGAN	91.77%	92.34%	90.44%	90.11%	91.23%	92.76%
Mask R-CNN	72.66%	82.81%	80.21%	75.42%	85.24%	78.58%

**Acc**_**o**_ = object level accuracy, * **Recall**_**o**_ = Object level recall, **Acc**_**p**_ = *Pixel level Accuracy, *Recall_**p**_ = Pixel level recall

### Comparison with state of the art

**Fig 11:**
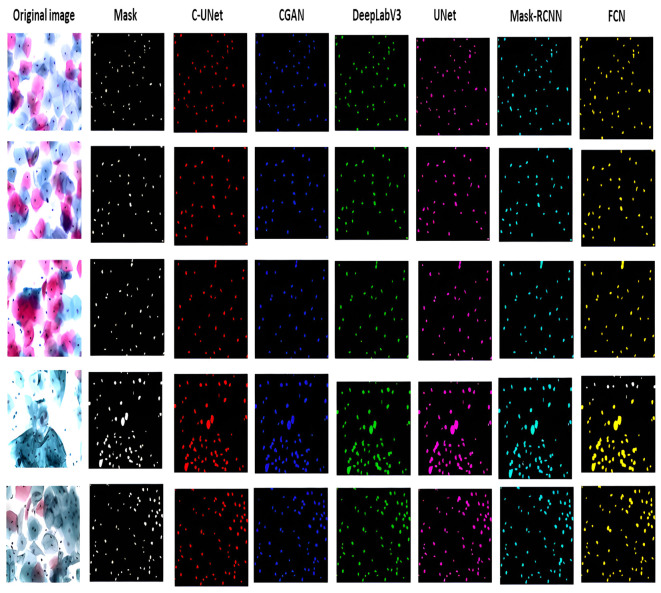
Shows cervical cell segmentation of state-of-the-art models.

We have compared the performance of the proposed C-UNet with state-of-the-art models employed for such tasks. To judge the accuracy of the models, a fivefold cross-validation scheme is adopted. Fully Connected Network (FCN) [60], CGAN [61], UNet [62], Mask RCNN [63], and deeplabv3 [64] were used. FCN employed VGG [65] as a basic network to extract features that perform slightly worse than other networks. By carefully examining Fig 11, we can observe that FCN suffers boundary leakage problems due to ambiguous boundary edges. UNet with U-shaped architecture to learn low and deep features performed slightly better than FCN but yielded over-segmentation when weak and strong edges appeared at the joining zones of clumped cervical cells. DeepLabv3 employed ResNet [66] to learn the features. DeepLabv3 is more appropriate for segmenting images with larger objects, while cervical nuclei are too small, and this network did not perform well for this task. CGAN represented a context-aware regressive network and employed gradient penalty for cervical nuclei identification and performed better than other networks but failed to split cell contours from the boundary.

Multiple cases include clumped nuclei, self-folding, intercellular overlapping, blurred contours, and diverse shapes of nuclei. As visualized in Fig 9, UNet detected nuclei from the cervical cells but failed to yield intact segmentation. Deeplabv3 performed better than others but was unable to identify impurities nuclei. CGAN proved more optimized than others for segmenting cervical cell nuclei but failed to identify nuclei and cytoplasm if they have similar colors. The proposed C-UNet outperformed them by segmenting background, nuclei, and impurities. The cervical smear images have a large proportion of small nuclei, high variations in resolution, blurry boundaries, and information features are low. The information related to nuclei is often contained in the inclusion, and this information mostly reduces due to continuous pooling and convolution operation. The C-UNet model uses 3 Bi-FPN layers that encompass direction connection layers in each BiFPN to prevent loss of significant features information. UNet uses interconnected decoders that merge boundary and semantic features and produce improved segmentation masks.

### Comparison with existing methods on ISBI datasets

We have considered the same evaluation metrics used in the prior studies for ISBI2014 and ISBI2015 to compare the performance of the proposed C-UNet. DSC, FNRo (False-negative rate at object level), FPRp (False positive rate at pixel level), and TPRp (True positive rate at pixel level) were used to measure the segmentation performance of the models in this section of the paper.

The nuclei segmentation performance of the existing methods and the proposed U-Net on the ISBI2014 dataset is listed in Table 3 below. [12] employed a three-pass fast watershed technique to segment the nucleus and cytoplasm from cervical cell scans. [23] integrated Voronoi diagrams and superpixels to segment cells into different parts. [28] developed a model for boundary approximation of clumped cells. They enhanced the boundary of cells by using the information in the stacks of images. [67] used a joint optimization of multi-level functions to distinguish cytoplasm and nuclei from clusters of clumped cervical cells. [14] developed a multiscale CNN model to distinguish cervical cell components. [68] designed a model based on contour refinement and partitioning of superpixel for cervical cell segmentation. We have selected these methods for fair comparison due to their best performance on the ISBI2014 and ISBI2015 datasets.

**Table 3:** Comparison of C-UNet with existing methods on ISBIs datasets using DSC (threshold > 0.7) TPRp, FNRo, and FPRp.

	DSC			TPRp			FNRo			FPRp		
Method	ISBI2014	ISBI2015	Dataset [44]	ISBI2014	ISBI2015	Dataset [44]	ISBI2014	ISBI2015	Dataset [44]	ISBI2014	ISBI2015	Dataset [44]
[12]	0.89 ± 0.07	0.85 ± 0.07	NA	0.91 ± 0.09	0.95 ±0.07	NA	0.27 ± 0.28	0.11 ± 0.17	NA	0.004± 0.005	0.004± 0.004	NA
[23]	0.87 ± 0.08	-	NA	0.90 ± 0.09	-	NA	0.14 ± 0.17	-	NA	0.005± 0.004	-	NA
[28]		0.85 ± 0.08	NA		0.94 ± 0.06	NA		0.16 ± 0.22	NA		0.005 ± 0.005	NA
[67]	0.88 ± N/A	-	NA	0.92 ± N/A	-	NA	0.21 ± N/A	-	NA	0.001± N/A	-	NA
[14]	-	0.89 ± N/A	NA	-	0.92 ± N/A	NA	-	0.26 ± N/A	NA	-	0.002± N/A	NA
[68]	0.90 ± 0.08	0.88 ± 0.09	NA	0.88 ± 0.10	0.88 ± 0.12	NA	0.14 ± 0.19	0.43 ± 0.17	NA	0.002± 0.003	0.001± 0.001	NA
[69]	0.93 ± 0.04	0.92 ± 0.05	NA	0.93 ± 0.05	0.93 ± 0.05	NA	0.91 ± 0.05	0.13 ± 0.15	NA	0.001 ± 0.002	0.001 ± 0.003	NA
C-UNet	**0.94 ± 0.04**	**0.93 ± 0.04**	**0.92± 0.04**	**0.94 ± 0.05**	**0.94 ± 0.05**	**0.96± 0.05**	**0.92 ± 0.05**	**0.12 ± 0.14**	**0.062±0.03**	**0.001 ± 0.002**	**0.001 ± 0.002**	**0.001± 0.002**
Standard UNet	NA	**NA**	**0.87± 0.05**	**NA**	**NA**	**0.90± 0.05**	**NA**	**NA**	**0.079±0.03**	**NA**	**NA**	**0.003± 0.003**
DeepLabv3	**NA**	**NA**	**0.90± 0.04**	**NA**	**NA**	**0.94± 0.05**	**NA**	**NA**	**0.69±0.03**	**NA**	**NA**	**0.002± 0.002**
FCN	**NA**	**NA**	**0.89± 0.05**	**NA**	**NA**	**0.93± 0.05**	**NA**	**NA**	**0.70±0.03**	**NA**	**NA**	**0.002± 0.002**

### Impact of loss functions on segmentation

To analyze the impact of different loss functions on the segmentation performance of the C-UNet, we have experimented with varying combinations of loss, and the results are summarized in Table 4. DSC, FNRo (False-negative rate at object level), FPRp (False positive rate at pixel level), and TPRp (True positive rate at pixel level) were used to measure the performance quantitatively. From Table 4, it is observed that the suggested loss combination and assigning values of *w*_*seg*_ and *w*_*bou*_ to 0.4 assists the proposed model in achieving optimum results than other combinations.

**Table 4:** Impact of various loss combinations for cervical cell segmentation on the C-UNet.

Segmentation loss	Boundary loss	DSC	FNRo	FPRp	TPRp
focal	Focal	0.76±0.09	0.35±0.17	0.002±0.003	0.75±0.07
Dice + BCE	Dice + BCE	0.90±0.07	0.25±0.16	0.001±0.003	0.90±0.06
Dice + BCE	Combo	0.89±0.07	0.27±0.17	0.001±0.003	0.88±0.08
Focal Tversky	Focal Tversky	0.79±0.08	0.34±0.18	0.002±0.003	0.80±0.07
Focal Tversky + *w*_*seg*_ (Dice+ BCE) w *w*_*seg*_ = 0.9	**FT+***w*_*bou*_ **Combo w** *w*_*bou*_ **= 0.9**	0.92±0.05	0.25±0.15	0.001±0.003	0.90±0.06
Focal Tversky + *w*_*seg*_ (Dice+ BCE) w *w*_*seg*_ = 0.4	**FT+***w*_*bou*_ **Combo w** *w*_*bou*_ **= 0.4**	0.93±0.04	0.24±0.14	0.001±0.003	0.91±0.06

### Impact of resolution on segmentation

In deep learning, input representation size and dataset volume are critical for optimum results. The original size of the cervical images in the employed dataset is 1024×768. We have experimented with different resolution patches, i.e., 256 x256, 512 x 512, and 768 x 768, to find the optimized size that can preserve spatial and contextual information. All experiments were conducted under identical conditions except for 768 x768 resolution, where batch size was set to 9 to minimize computation cost. We have observed optimum performance in the case of 512 x 512, as shown in Table 5.

**Table 5:** Shows the segmentation results with different patch size.

Resolution	Acc_o_	Recall_o_	Acc_p_	Recall_p_	Dice Coefficient	F1-Score
768 x768	89.26%	90.23%	89.22%	90.02%	89.03%	89.62%
512 x 512	93.00%	95.32%	92.56%	92.27%	93.12%	94.96%
256 x256	90.09%	91.42%	89.66%	89.86%	88.12%	90.03%

### Impact of data augmentation

Table 6.

**Table 6:** Shows the impact of data augmentation on the proposed network performance.

Method	Augmentation	Acc_o_	Recall_o_	Acc_p_	Recall_p_	Dice Coefficient	F1-Score
C-UNet	Yes	93.00%	95.32%	92.56%	92.27%	93.12%	94.96%
C-UNet	No	87.29%	88.40%	86.61%	85.86%	84.82%	88.17%

## Conclusion

In this paper, we have proposed a deep neural network based on the architecture of classic UNet to improve the nucleus segmentation from cervical smear images. We have designed a cross-scale feature integration module to preserve the spatial and local features on the basis of their significance. We have incorporated a wide context unit into the baseline UNet to extract the contextual information and feature accumulation at the transition level in order to enhance nucleus segmentation. A decoder was composed of two inter-connecter decoders to generate segmentation and boundary masks. The proposed C-UNet was evaluated on the cervical smear images dataset, ISBI2014, and ISBI2015 datasets. To enhance the training of the proposed model, we have employed stain normalization, other data augmentation, and transformation techniques. The evaluation of the model has shown the performance of the proposed model is superior to those of the extant models. Although, C-UNet has shown an improvement in the segmentation of cervical nuclei segmentation, pathologist confirmation may be needed in a practical setting. This network is expensive computationally because of the C-UNet depth and inter-connected decoders. In the future, we will focus on minimizing computational overhead, investigating contrastive learning to enhance model performance, and generalizing for other applications.
